# Supplementary material for: dnmt1 function is required to maintain retinal stem cells within the ciliary marginal zone of the zebrafish eye
Source: Sci Rep. 2020 Jul 9;10:11293. doi: 10.1038/s41598-020-68016-z (PMC7347529; doi:10.1038/s41598-020-68016-z)
Supplement: Supplementary file 1 — Supplementary information [file 41598_2020_68016_MOESM1_ESM.docx]

***dnmt1* function is required to maintain retinal stem cells within the ciliary marginal zone of the zebrafish eye ­**

Krista M. Angileri^1^ and Jeffrey M. Gross^1,2^*

^1^: Department of Ophthalmology, Louis J. Fox Center for Vision Restoration, University of Pittsburgh School of Medicine, Pittsburgh, PA

^2^: Department of Developmental Biology, University of Pittsburgh School of Medicine, Pittsburgh, PA

***Corresponding author:** Jeffrey M. Gross, Department of Ophthalmology, Louis J. Fox Center for Vision Restoration, University of Pittsburgh School of Medicine, Pittsburgh PA, 15213, USA; [grossjm@pitt.edu](mailto:grossjm@pitt.edu)

**­**

***Supplementary Information:***


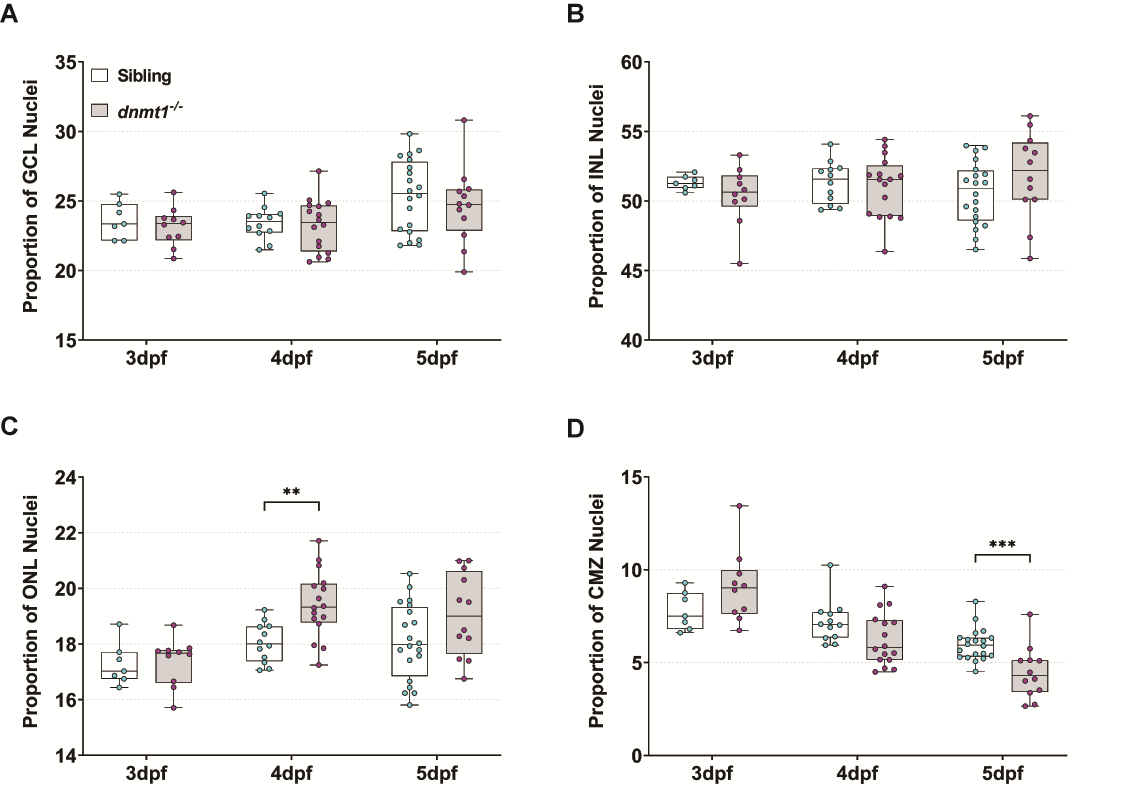


**Supplementary Figure S1. Loss of dnmt1 function results in a decline of RSC number.** **A-D.** Graphs of retinal domain proportions over time between siblings and *dnmt1^-/-^* larvae. ***p* <0.005, *** = *p* <0.0005.


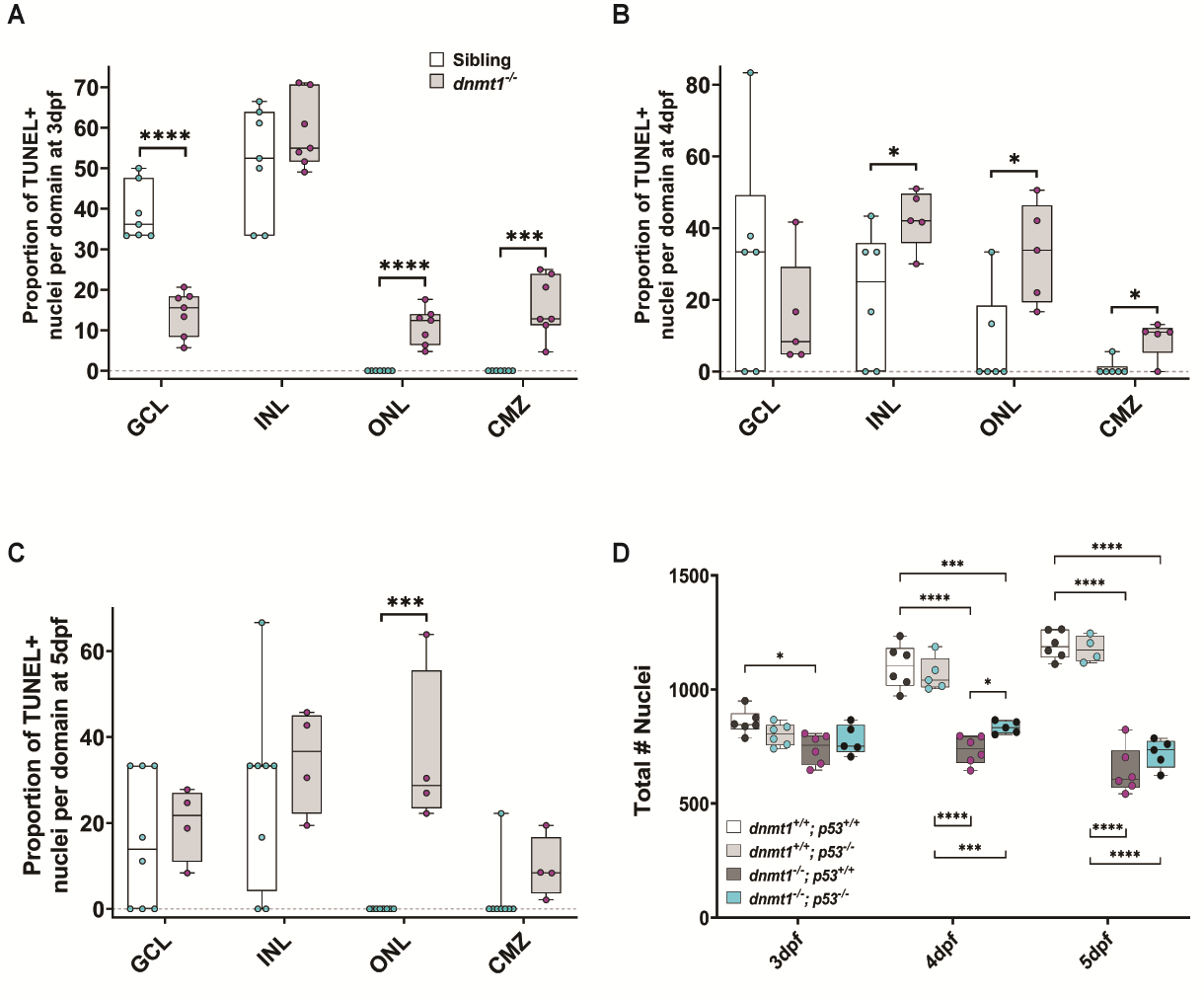


**Supplemental Figure S2. p53-mediated apoptosis is not responsible for *dnmt1^-/-^* RSC loss. A-C.** Proportion of TUNEL^+^ nuclei in each retinal domain of *dnmt1^-/-^* larvae (gray bars) compared to sibling controls (white bars) at 3dpf (**A**), 4dpf (**B**), and 5dpf (**C**). **D.** Total number of retinal nuclei between *dnmt1^+/+^;p53^+/+^* (white bars), *dnmt1^+/+^;p53^-/-^* (light gray bars), *dnmt1^-/-^*;*p53^+/+^* (dark gray bars), *dnmt1^-/-^*;*p53^-/-^* (blue bars) larvae from 3-5dpf. GCL: ganglion cell layer; INL: inner nuclear layer; ONL: outer nuclear layer; CMZ: ciliary marginal zone. **p* <0.05, ****p* <0.0005, *****p* <0.00005.


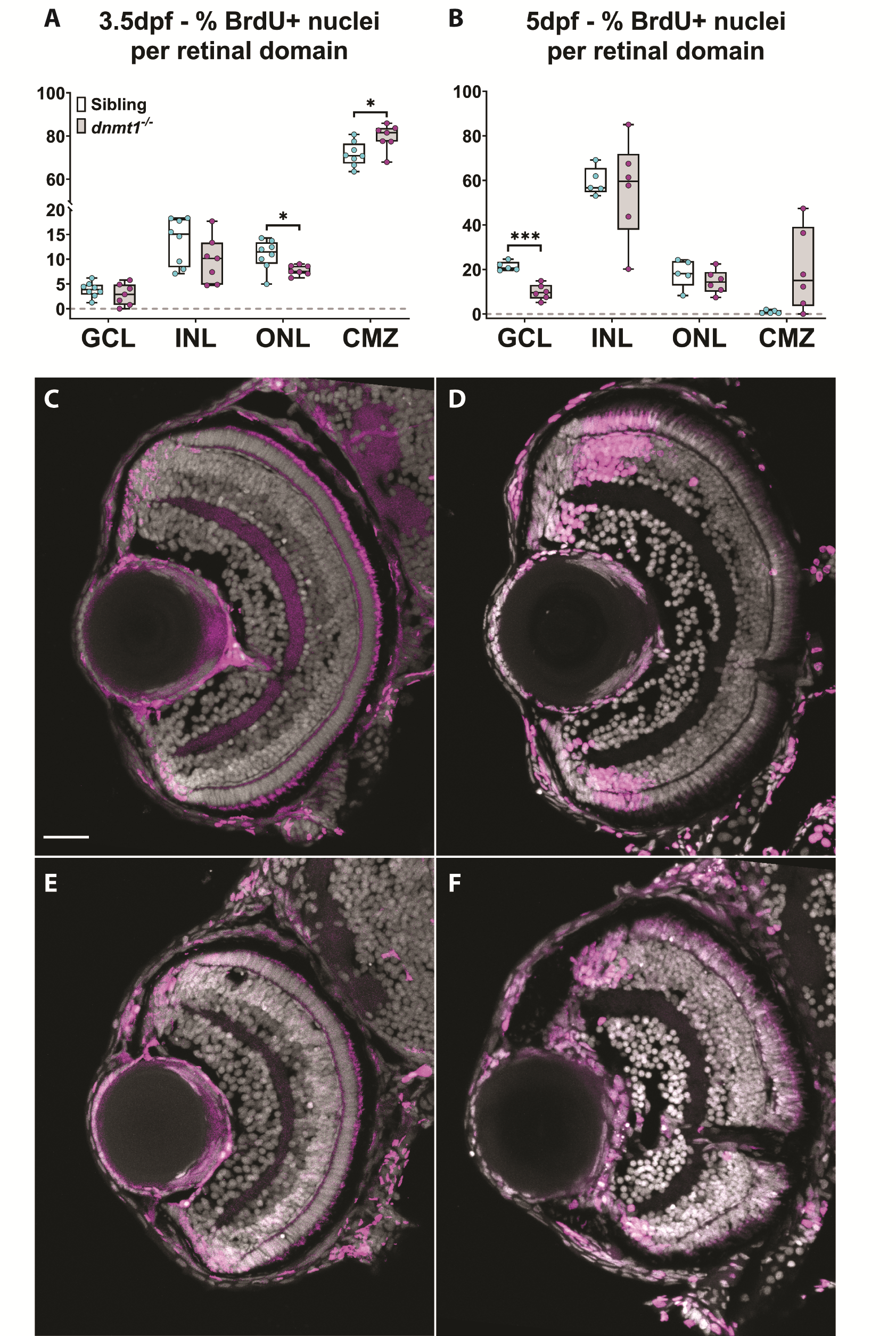


**Supplemental Figure S3. dnmt1-deficient RSCs fail to incorporate into the neural retina.** **A.** Data points collected of the proportion of cells labeled with BrdU in each retinal domain at 3.5dpf divided by the number of total BrdU^+^ cells. **B.** Data points collected of the proportion of cells labeled with BrdU in each layer at 5dpf divided by the number of total BrdU^+^ cells. Sibling controls = white bars; *dnmt1^-/-^* = gray bars. **p* <0.05, ****p* <0.0005. **C-F.** Transverse sections of sibling (C-D) and *dnmt1^-/-^* (E-F) retinae at 3.5dpf (C,E) and 5dpf (D,F). Nuclei labeled with DAPI (gray). BrdU^+^ cells (magenta). Dorsal is up in all images. Scale bar (C) = 30 μm. All images taken at the same magnification.


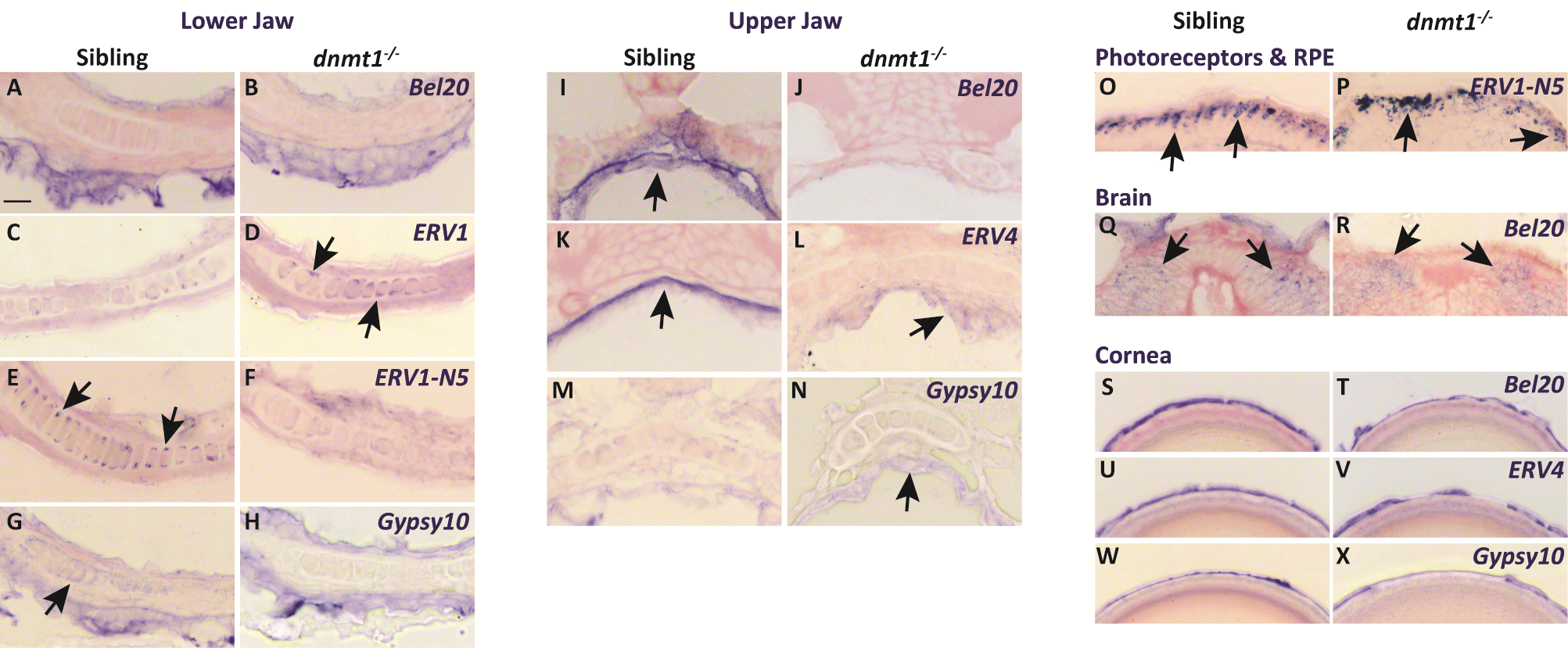


**Supplemental Figure S4. Loss of dnmt1 function results in misregulation of LTR RE expression across numerous tissues.** **A-X.** Transverse cryosections of larvae analyzed by *in situ* hybridization. All sibling and *dnmt1^-/-^* larvae are 4dpf. **A-H.** Expression of indicated REs within the lower jaw. **I-N.** Expression of indicated REs within the upper jaw. **O-P.** Expression of *ERV1-N5 LTR* in photoreceptors (**O**; sibling) and the RPE (**P**; *dnmt1^-/-^*). **Q-R.** Expression of *Bel20 LTR* within the brain. **S-X.** Expression of indicated REs within the cornea. Arrows delineate expression changes of specified REs. Scale bar (A) = 10 μm. All images taken at the same magnification. RPE = retinal pigmented epithelium.


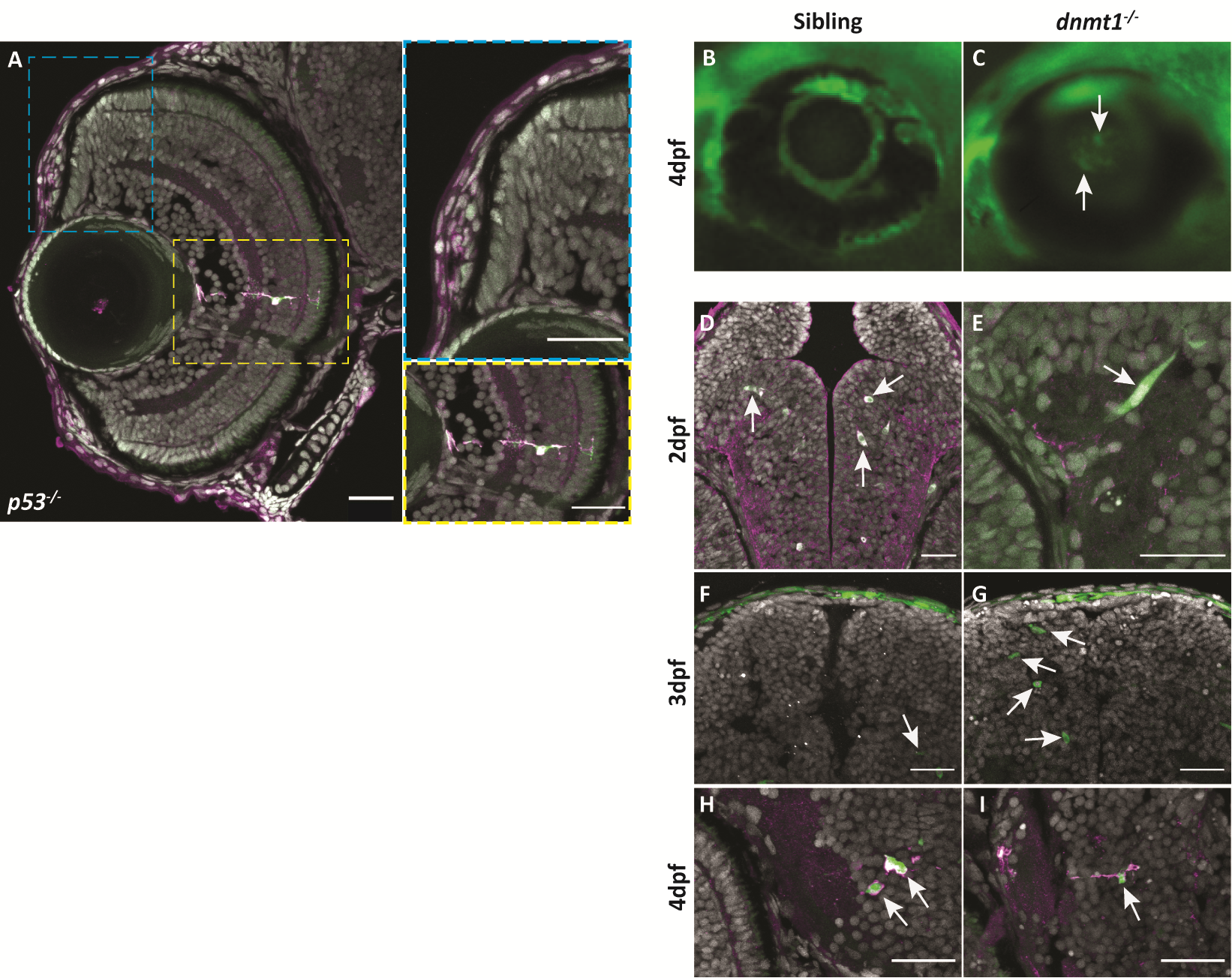


**Supplemental Figure S5. L1RE3-EGFP transgene expression is more prominent in *dnmt1^-/-^* larvae.** **A-A’’.** Transverse section of *Tg(CMV:Hsa.L1RE3,EGFP,myl7:EGFP;p53^-/-^)* larvae at 4dpf. **A.** L1RE3-EGFP^+^ retinal cells labeled with endogenous EGFP. Cyan (A‘) and yellow (A’‘) dotted boxes indicate magnified images to the right of panel A. **A’.** Magnified image of *Tg(CMV:Hsa.L1RE3,EGFP,myl7:EGFP;p53^-/-^)* CMZ showing no expression of the L1RE3-EGFP transgene. **A’’.** Magnified image of retinal neuron expressing L1RE3-EGFP transgene. **B-I.** All images are taken from *Tg(CMV:Hsa.L1RE3,EGFP,myl7:EGFP)* larvae that are either *dnmt1^+/+^* (B,D,F,H) or *dnmt1^-/-^* (C,E,G,I). **B-C.** Whole-mount images of 4dpf eyes demonstrating L1RE3-EGFP transgene activation seen through the lens of *dnmt1^-/-^* larvae and not siblings. **D,F,H.** Sibling larvae expressing the L1RE3-EGFP transgene in the brain. **E,G,I.** *dnmt1^-/-^* larvae expressing the L1RE3-EGFP transgene in the brain. Nuclei labeled with DAPI (gray). Endogenous L1RE3-activated EGFP labeled in green. EGFP antibody is magenta. Scale bars: 30 μm in all images. Dorsal is up in all images.

**Supplementary Table S1. List of primer sequences and appropriate experimental purpose used in this study.**

| ***Gene / Target*** | **Fwd Primer Sequence** | **Rev Primer Sequence** | **Experimental Purpose** |
| --- | --- | --- | --- |
| *dnmt1^s872^* | GAATCAATGCACACCATATGCCTTATGTAC | CTCAGGGTGAAGGACACGTCC | HRM Genotyping TL strain |
| *dnmt1^s872^* | AATGCTGTTCCTCCACCCC | CTTGACCTCCAGGCCAATGG | HRM Genotyping AB strain |
| *tp53^zdf1^* | CTAAACTACATGTGCAATAGCAGCTGC | CTCCAGAGTGATGATTGTGAGGATGG | HRM Genotyping |
| *ef1α* | GTACTTCTCAGGCTGACTGTG | ACGATCAGCTGTTTCACTCC | qPCR Analysis Control |
| *β-actin* | AAGCAGGAGTACGATGAGTCTG | GGTAAACGCTTCTGGAATGAC | qPCR Analysis Control |
| *gapdh* | GTGGAGTCTACTGGTGTCTTC | GTGCAGGAGGCATTGCTTACA | qPCR Analysis Control |
| *ccna2* | ACGAGACTCTTTACCTGGCT | GAGAGAACTGTCAGCACCAG | qPCR Analysis |
| *ccnb1* | TGACATGGTCCACTACCCTC | GATGCTTAGAAAGGCCCTCG | qPCR Analysis |
| *ccnd1* | AAGTGGGATCTGGCCTCAGT | GGCAACTGTCGGTGCTTTTC | qPCR Analysis |
| *ccne* | GGACTGCGGAACACATCAC | CGGTTCCTCGACTTCATCAG | qPCR Analysis |
| *cdk1* | GCTTCACGCTATTCCACACC | GCCAGATTCCCAGATTTCCAC | qPCR Analysis |
| *cdk2* | GACTACAAACCCTCCTTTCCC | AAACCGATGAACAAGAGCGT | qPCR Analysis |
| *cdk4* | GCAGTATGAGCCAGTAGCAG | ATGTTGGGATGGTCGAACTG | qPCR Analysis |
| *cdkn1a* | TAGACGCTTCTTGGCTTGGT | ATCCCGAAAACACCAGAACG | qPCR Analysis |
| *cdkn1ba* | TCAGCACGCCGAGGAAACGA | CTGGCGAAGTAGTCGATGGTGAG | qPCR Analysis |
| *cdkn1ca* | AGGCGATTTCAGAGGACACTTTGC | GGAAGCGTCTCCTGTTGCGTTAA | qPCR Analysis |
| *dnmt1* | AAGAACGGCTTCAGGTGTTG | CGTCTCCTTCCTCCTGAGTG | qPCR Analysis |
| *caspa* | AAAAGGAGCGGCTCAGAGAA | CACCCATAATGGCGTCTCTT | qPCR Analysis |
| *caspb* | GGCTGCTGAGATCTCTGGGCTTCGC | CTTCCCTCGATTGGATTCCTGTATGCG | qPCR Analysis |
| *mdm2* | CCCTCACTGCGAGATATACC | GAGGCATAAGTCGGACAGCT | qPCR Analysis |
| *p53* | GGTGCTGAATGGACAACTGTGCT | GCAACTGACCTTCCTGAGTCTCC | qPCR Analysis |
| *ripk1* | GCCGTATGAGAATGCAAGAAGTGAG | CCGATTATAGCAATCTGTGAAGGAGGG | qPCR Analysis |
| *tnf-α* | ACCAGGCCTTTTCTTCAGGT | GCATGGCTCATAAGCACTTGTT | qPCR Analysis |
| *il-1β* | TTCCCCAAGTGCTGCTTATT | AAGTTAAAACCGCTGTGGTCA | qPCR Analysis |
| *BEL20-LTR_DR* | AATGCAACGCAGTATCATCG | AGGTGCACTTCTCCGAGTGT | *In situ* hybridization probe |
| *ERV1-1-LTR_DR* | TGAAGGATTTGATCTTCTCTCC | GCAATCGAGTCAGTGGGTCT | *In situ* hybridization probe |
| *ERV1-N5-LTR_DR* | TGAGTATGCCTGCACAGGAG | TCAGACCGGAGGTTTTGAAT | *In situ* hybridization probe |
| *ERV4_DR-I* | CCAAGACCGATCACACCTTT | ACTCCCATAATTCCCCCTTG | *In situ* hybridization probe |
| *Gypsy10-LTR_DR* | TGCGGTTAACGCTTACAAAA | CACTCCCCCTAATCAGATACCA | *In situ* hybridization probe |
